# Gut microbiota composition and function in pregnancy as determinants of prediabetes at two-year postpartum

**DOI:** 10.1007/s00592-023-02064-5

**Published:** 2023-04-28

**Authors:** Noora Houttu, Chouaib Benchraka, Mrunalini Lotankar, Ella Muhli, Harri Niinikoski, Leo Lahti, Kirsi Laitinen

**Affiliations:** 1 Institute of Biomedicine, Research Centre for Integrative Physiology and Pharmacology, University of Turku, Turku, Finland; 2 Department of Computing, Faculty of Technology, University of Turku, Turku, Finland; 3 Department of Obstetrics and Gynecology, University of Turku, Turku, Finland; 4 Department of Pediatrics and Adolescent Medicine, Turku University Hospital, Turku, Finland; 5 Functional Foods Forum, University of Turku, Turku, Finland

**Keywords:** Postpartum prediabetes, Gut microbiota during pregnancy, Metagenomics, Prospective study

## Abstract

**Aims:**

Deep metagenomics offers an advanced tool for examining the relationship between gut microbiota composition and function and the onset of disease; in this case, does the composition and function of gut microbiota during pregnancy differ in women who develop prediabetes and those who do not at two-year postpartum, and whether the gut microbiota composition associates with glycemic traits.

**Methods:**

In total, 439 women were recruited in early pregnancy. Gut microbiota was assessed by metagenomics analysis in early (13.9 ± 2.0 gestational weeks) and late pregnancy (35.1 ± 1.0 gestational weeks). Prediabetes was determined using American Diabetes Association criteria as fasting plasma glucose 5.6–6.9 mmol/l analyzed by an enzymatic hexokinase method. Of the women, 39 (22.1%) developed prediabetes by two-year postpartum.

**Results:**

The relative abundances of *Escherichia unclassified* (FDR < 0.05), *Clostridiales bacterium* 1_7_ 47FAA (FDR < 0.25) and *Parabacteroides* (FDR < 0.25) were higher, and those of *Ruminococcaceae bacterium* D16 (FDR < 0.25)*, Anaerotruncus unclassified* (FDR < 0.25) and *Ruminococcaceae noname* (FDR < 0.25) were lower in early pregnancy in those women who later developed prediabetes. In late pregnancy, *Porphyromonas* was higher and *Ruminococcus* sp 5_1_39BFAA was lower in prediabetes (FDR < 0.25). Furthermore, fasting glucose concentrations associated inversely with *Anaerotruncus unclassified* in early pregnancy and directly with *Ruminococcus* sp 5_1_39BFAA in late pregnancy (FDR < 0.25)*. α*-Diversity or *β*-diversity did not differ significantly between the groups. Predictions of community function during pregnancy were not associated with prediabetes.

**Conclusions:**

Our study shows that some bacterial species during pregnancy contributed to the onset of prediabetes within two-year postpartum. These were attributable primarily to a lower abundance of short-chain fatty acids-producing bacteria.

**Supplementary Information:**

The online version contains supplementary material available at 10.1007/s00592-023-02064-5.

## Introduction

The gut microbiota composition has been associated with several metabolic diseases such as gestational diabetes mellitus (GDM) [1] and type 2 diabetes mellitus (T2D) [2], but the role of the gut microbiota in the onset of these diseases is not completely clear. Prediabetes is a state in which glucose and insulin homeostasis is impaired, but the diagnostic criteria for T2D are not met. Pregnant women with overweight and obesity are an especially important group of individuals because of their increased risk to develop GDM, which in turn predisposes to the development of T2D [3].

Recently, it has been postulated that gut microbiota may predict the incidence of T2D [4] and relate to prediabetes [5–7], as studied previously primarily by 16S sequencing. Nonetheless, there are much fewer studies which have applied a metagenomics approach, even though this allows a deeper evaluation of gut microbiota composition and also a prediction of the functional profile; furthermore, these have focused on non-pregnant adults [8–11]. These studies indicate that the abundance of several butyrate-producing bacteria, e.g., *Faecalibacterium* spp., [8, 9] and the abundance of genes linked with butyrate production [8] were decreased in subjects with prediabetes as compared to subjects with normal-glucose control.

To our knowledge, there are no prior studies examining the relationship of the gut microbiota composition and function during pregnancy on the onset of prediabetes at postpartum. Therefore, this study’s first objective was to investigate whether the gut microbiota composition and function during pregnancy differ in women who develop prediabetes and those who do not at two-year postpartum. Secondly, we assessed the associations between gut microbiota, prepregnancy body mass index (BMI), high-sensitivity C-reactive protein (hs-CRP) and glycemic traits. Since the gut microbiota characteristics may change during the course of the pregnancy [12, 13], we examined two time points: one in early and the other in late pregnancy. By identifying alterations in gut microbiota composition already in the early stages of the march toward diabetic disease, this might help us to understand the role of gut microbiota in the pathology of these diseases and thus may offer new tools for their detection and prevention.

## Material and methods

### Study design and subjects

This single-center mother-infant trial (ClinicalTrials.gov: NCT01922791) was conducted in Turku, Southwest Finland, and the study subjects were recruited (*n* = 439) between 2013 and 2016. The study complied with the Declaration of Helsinki as revised in 2000. The Ethics Committee of the Hospital District of Southwest Finland approved the study protocol, and all participants provided written informed consent. The study has been described in detail previously [14]. Briefly, the inclusion criteria were overweight (BMI ≥ 25 kg/m^2^) and early pregnancy (< 18 gestational weeks) and absence of chronic diseases. The exclusion criteria were diabetes before pregnancy (HbA1c ≥ 6.5% [48 mmol/mol] or fasting glucose ≥ 7.0 mmol/L at randomization), twin pregnancy, and chronic diseases influencing metabolic and gastrointestinal health. The main trial investigated the effect of fish oil and/or probiotics on maternal and child health, the primary outcomes being glucose metabolism during pregnancy and allergy in child.

**Fig. 1:**
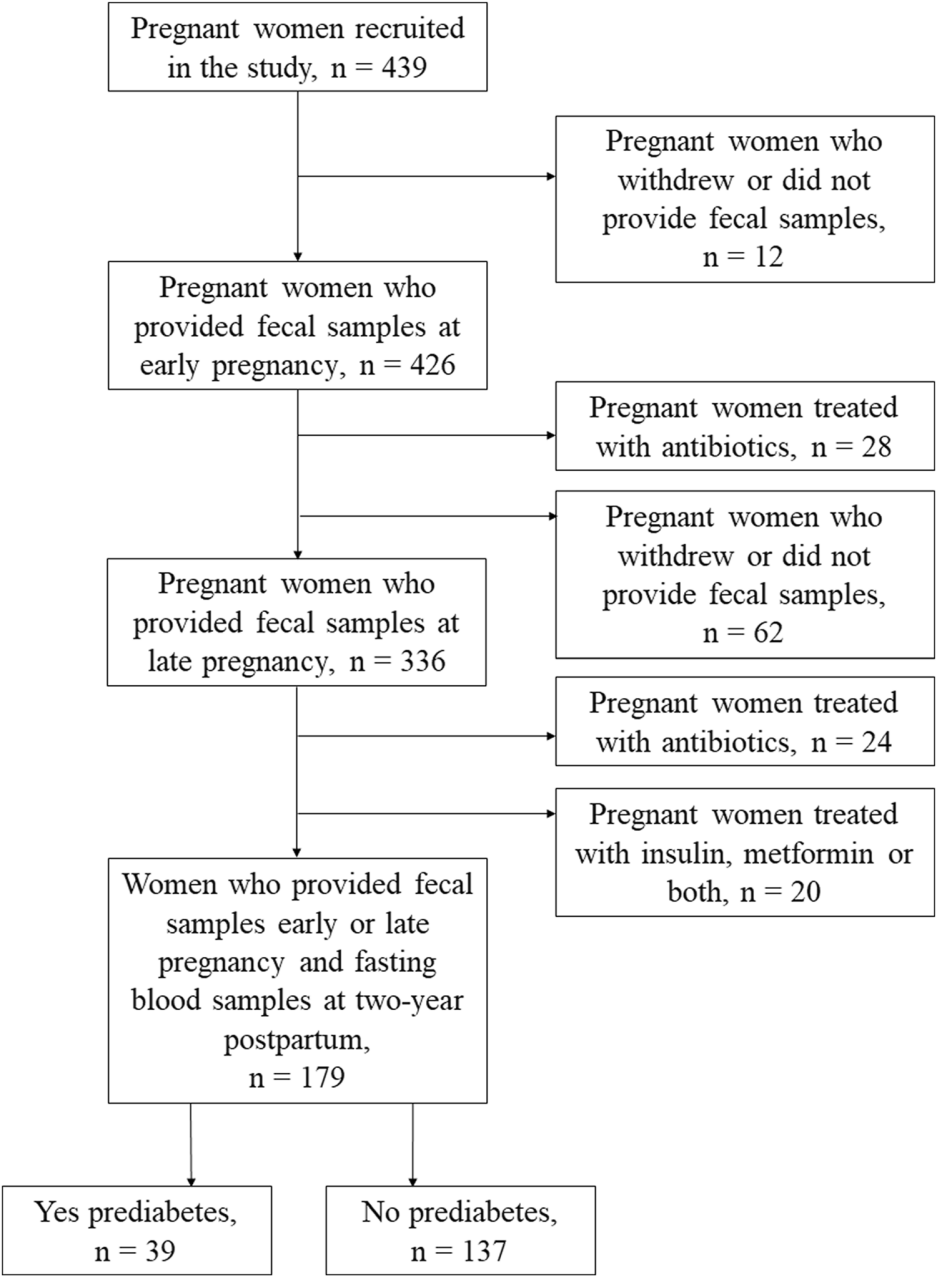
The flowchart of the present study

In the present study, we evaluated the relationship between the gut microbiota in early and late pregnancy and the incidence of prediabetes at two-year postpartum. Women who had used antibiotics within eight weeks before fecal sampling and women who did not provide a fecal sample in either early or late pregnancy or were treated with metformin or insulin in late pregnancy were excluded. A total of 176 women, 39 belonging to the group of women developing prediabetes at two-year postpartum and 137 to the group of women who did not develop prediabetes at two-year postpartum were included into this study (Fig. 1, flowchart).

### Prediabetes definition and clinical parameters

The condition of prediabetes was determined when the individual displayed a fasting plasma glucose concentration in the range 5.6–6.9 mmol/l according to American Diabetes Association criteria [15]. On the morning of the study visit, after at least 9 h of overnight fasting, blood samples were drawn from an antecubital vein. The fasting plasma glucose was analyzed by an enzymatic method using hexokinase (Cobas 8000 automatic c702-analyzer, Roche Diagnostics GmbH, Mannheim, Germany) in early pregnancy and two-year postpartum. In early pregnancy and two-year postpartum, insulin concentrations were determined with an immunoelectrochemiluminometric assay on a modular E170 automatic analyzer (Roche Diagnostics GMbH, Mannheim, Germany) and HbA_1c_ was measured by ion-exchange HPLC by the Bio-Rad Variant II Haemoglobin A1c Program (Bio-Rad Laboratories, Marnes-la-Coquette, France) and insulin resistance by homeostasis model assessment (HOMA2-IR) [16]. High-sensitivity C-reactive protein (hs-CRP) was determined by an automated colorimetric immunoassay on the Dade Behring Dimension RXL autoanalyzer (Siemens Healthcare, Camberly, Surrey, UK). Blood pressure was measured with Omron M5-1 (IntelliTM sense, Omron Matsusaka Co., Ltd, Japan). Diet intake was calculated from 3-day food diaries by computerized software (AivoDiet 2.0.2.3, Aivo, Turku, Finland) utilizing the Finnish Food Composition Database Fineli [17]. Prepregnancy BMI (kg/m^2^) was calculated. Self-reported prepregnancy weight was obtained from the maternal welfare clinic records and height measured in early pregnancy using a wall stadiometer in 0.1 cm accuracy. The women filled in a questionnaire about their clinical background information. Information on antibiotic usage was inquired from a question in the diary and confirmed by interview in the study visits.

### Fecal sampling and analyses

Fecal samples were collected in sterile plastic pots on the morning of the study visit or the previous evening in early and late pregnancy and kept at − 20 °C until DNA extraction. The details of DNA extraction as well as metagenomics and functional analyses have been described in supplementary material.

### Bioinformatics and statistical analyses

Bioinformatics were performed using R version 4.2.1. The source code for the analyses is available online [18]. Community composition was compared between the prediabetes groups with respect to α-diversity, β-diversity, and differential abundance. These analyses were adjusted for prepregnancy BMI and the early or late pregnancy daily dietary intake of polyunsaturated fatty acids (PUFA) since they were associated with the prediabetes status (Mann–Whitney *p* = 0.01; independent samples *T*-test, *p* = 0.02, respectively). The intervention was not included as a covariate in the models since the intervention group was not associated with the prediabetes status (*Χ*^2^, *p* = 0.46) and increasing the number of covariates would reduce the statistical power of the tests. The analyses were corrected for multiple comparisons using the function *stats::p.adjust* (Benjamini–Hochberg FDR method). *p* < 0.05 and FDR < 0.25 for differential abundance and Spearman correlation were considered significant, respectively. *α*-Diversity was compared between the prediabetic and non-prediabetic groups by using a linear model. The overall differences in taxonomic composition (i.e., *β*-diversity) were quantified with Bray–Curtis dissimilarity and visualized with Principal Coordinates Analysis (PCoA) using the *mia* [19] and *miaViz* R packages. Associations between gut microbiota composition and prediabetes status were evaluated with PERMANOVA from the R *vegan* package [20] with the *vegan::adonis* function, checked for the homogeneity condition with the *vegan:: betadisper* function and test with *stats::anova* from the *stats* package [21]. Differential abundances at the genus and species levels were tested with MaAsLin 2 [22, 23] with the function *maaslin2::Maaslin2,* using the original relative abundance data. The predictability of functional data to the prediabetes status was evaluated with Random Forest with the R *ranger* package [24] using fivefold cross-validation with the *caret* R package [25]. Spearman correlation was used to quantify associations between bacterial species and genus abundances and prepregnancy BMI, hs-CRP and glycemic traits (fasting glucose, insulin, HbA_1c_, HOMA2-IR) for those bacterial taxa that showed significant differential abundance between the prediabetes groups.

Continuous baseline clinical variables defining the characteristics of the women analyzed by SPSS Statistics 24.0 (IBM, Chicago, IL, USA) and the differences between women and those not developing prediabetes were tested with independent samples *T*-test if the variables were normally distributed whereas if the variables were non-normally distributed, the Mann–Whitney test was applied. The differences in categorical clinical baseline variables between the groups were tested with *Χ*^2^ or Fisher’s exact test.

## Results

### Clinical characteristics

The baseline clinical characteristics of the women are presented in Table 1. Women developing prediabetes had a higher prepregnancy BMI (30.5 (27.7–34.1) kg/m^2^ vs 28.4 (26.2–31.0) kg/m^2^, *p* = 0.01) and they consumed more PUFA (14.7 ± 6.2 g) than those who did not develop prediabetes (12.6 ± 4.7 g, *p* = 0.02). Thus, prepregnancy BMI and daily dietary intake of PUFA at baseline were included as confounding factors for early pregnancy, and the relevant variables were also included as confounding factors in the late pregnancy analyses. The number of women developing prediabetes did not differ between the intervention groups (data not shown).

**Table 1:** Clinical characteristics of the study subjects in early pregnancy

Clinical characteristics	Prediabetes	No prediabetes	All	*n*	*P* value
Age (y)^a^	32.8 ± 4.3	31.0 ± 4.7	31.4 ± 4.6	39/137/176	**0.03**
Education (college or university degree) (*n*, %)^c^	22, 56.4	93, 67.9	115, 65.3	39/137/176	0.18
Ethnicity (*n*, %)^c^				39/137/176	1.00
European	39, 100	134, 97.8	173, 98.3		
Asian	0, 0	1, 0.7	1, 0.6		
Other/mixed	0, 0	2, 1.5	2, 1.1		
Smoking before pregnancy (*n*, %)^c^				39/137/176	0.36
Yes	9, 23.1	23, 16.8	32, 18.2		
No	30, 76.9	114, 83.2	144, 81.8		
Prepregnancy BMI (kg/m^2^)^d^	30.5 (27.7–34.1)	28.4 (26.2–31.0)	28.9 (26.5–31.4)	39/137/176	**0.01**
Systolic blood pressure (mmHg)^d^	119.0 (113.5–124.5)	116.5 (110.3–125.0)	117.5 (111.0–125.0)	39/136/175	0.18
Diastolic blood pressure (mmHg)^a^	80.2 ± 8.5	77.4 ± 8.3	78.0 ± 8.4	39/136/175	0.07
GDM (*n*, %)^be^				38/133/171	** < 0.001**
Yes	23, 60.5	26, 19.5	49, 28.7		
No	15, 39.5	107, 80.5	122, 71.3		
Insulin (mU/l)^d^	11.0 (8.0–13.3)	9.0 (7.0–14.0)	10.0 (8.0–14.0)	38/135/173	0.14
Glucose (mmol/l)^a^	4.9 ± 0.4	4.7 ± 0.3	4.8 ± 0.4	38/135/173	**0.02**
HOMA2-IR^d^	1.4 (1.0–1.7)	1.2 (0.9–1.8)	1.3 (1.0–1.8)	38/135/173	0.11
HbA_1c_ (mmol/mol)^a^	30.7 ± 3.2	29.3 ± 2.9	29.6 ± 3.0	38/133/171	**0.008**
HbA_1c_ (%)^a^	5.0 ± 0.3	4.8 ± 0.3	4.9 ± 0.3	38/133/171	**0.009**
History of GDM (*n*, %)^c^				39/137/176	0.52
Yes	2, 5.1	13, 9.5	15, 8.5		
No	37, 94.9	124, 90.5	161, 91.5		
Family history of diabetes (*n*, %)^c^				39/136/175	0.79
Yes	9, 23.1	27, 19.9	36, 20.6		
No	29, 74.4	101, 74.3	130, 74.3		
Does not know	1, 2.6	8, 5.9	9, 5.1		
Alcohol usage (*n*, %)^c^				39/137/176	0.36
Never	35, 89.7	126, 92.0	161, 91.5		
Once in a month or less frequently	2, 5.1	9, 6.6	11, 6.3		
2–4 times in a month	2, 5.1	2, 1.5	4, 2.3		
Dietary intake					
Energy (kJ)^a^	8929.5 ± 2356.8	8277.8 ± 1904.9	8426.4 ± 2028.1	39/132/171	0.07
Protein (g)^a^	84.2 ± 21.0	78.8 ± 20.5	80.0 ± 20.7	39/132/171	0.15
Carbohydrate (g)^a^	230.4 ± 67.1	222.4 ± 58.9	224.2 ± 60.7	39/132/171	0.47
Fat (g)^d^	87.5 (71.6–109.4)	79.4 (62.9–96.4)	80.8 (64.2–98.0)	39/132/171	0.05
Saturated fat (g)^d^	31.3 (22.7–40.8)	27.4 (22.3–35.1)	28.1 (22.5–35.9)	39/132/171	0.19
Monosaturated fat (g)^d^	29.1 (25.3–37.0)	26.0 (20.7–33.8)	26.6 (20.9–34.2)	39/132/171	0.05
Polyunsaturated fat (g)^a^	14.7 ± 6.2	12.6 ± 4.7	13.1 ± 5.2	39/132/171	**0.02**
Fiber (g)^d^	20.0 (14.5–25.0)	20.8 (16.2–26.3)	20.7 (15.2–26.1)	39/132/171	0.59
Protein (E%)^a^	16.3 ± 3.0	16.3 ± 3.2	16.3 ± 3.1	39/132/171	0.99
Carbohydrate (E%)^a^	43.9 ± 6.5	45.8 ± 6.3	45.3 ± 6.3	39/132/171	0.11
Fat (E%)^a^	37.4 ± 6.6	35.5 ± 6.2	35.9 ± 6.4	39/132/171	0.10
Saturated fat (E%)^a^	13.2 ± 3.1	13.0 ± 3.0	13.0 ± 3.1	39/132/171	0.71
Monosaturated fat (E%)^a^	12.8 ± 3.2	12.2 ± 2.8	12.3 ± 2.9	39/132/171	0.26
Polyunsaturated fat (E%)^d^	5.9 (4.8–6.6)	5.5 (4.4–6.5)	5.6 (4.6–6.6)	39/132/171	0.13

^a^Independent samples *T*-test

^b^*Χ*^2^

^c^Fisher’s exact test

^d^Mann–Whitney

^e^GDM was diagnosed in 12–16 or 24–28 gestational weeks on the basis of a 2-h 75 g oral glucose tolerance test if one or more values were: 0 h ≥ 5.3, 1 h ≥ 10.0 and 2 h ≥ 8.6 mmol/L according to Finnish Current Care guidelines (Gestational Diabetes Mellitus. Current Care Guidelines. Working group set by the Finnish Medical Society Duodecim, the Medical Advisory Board of the Finnish Diabetes Association and the Finnish Gynecological Association. Helsinki: The Finnish Medical Society Duodecim, 2022 (referred January 11, 2023). Available online at: www.kaypahoito.fi)

*p* < 0.05 is considered statistically significant

### Determinants of prediabetes at postpartum: gut microbiota diversity during pregnancy

*α*-Diversity (Shannon index, Suppl. Fig. S1) in early or late pregnancy did not differ between women developing and those not developing prediabetes (early pregnancy *p* = 0.12, late pregnancy *p* = 0.75; linear model). Similarly, no difference could be visually observed in *β*-diversity between the two groups as visualized by PCoA using Bray–Curtis dissimilarity (Suppl. Fig. S2). Although no distinct groups were evident, the PERMANOVA analysis revealed that the gut microbiota composition was influenced by the dietary intake of PUFA in early (*p* = 0.03) but not in late pregnancy (*p* = 0.26) (Suppl. Fig. S3). When the test was conducted separately for each prediabetes group and pregnancy stage, the intake of PUFA was only significant in early pregnancy in those women developing prediabetes (*p* = 0.01).

### Determinants of prediabetes at postpartum: abundance of bacterial genera and species during pregnancy

A total of 150 species and 55 genera were identified in the fecal samples collected during pregnancy (Suppl. Methods, Suppl. Table S1-2).

**Fig. 2:**
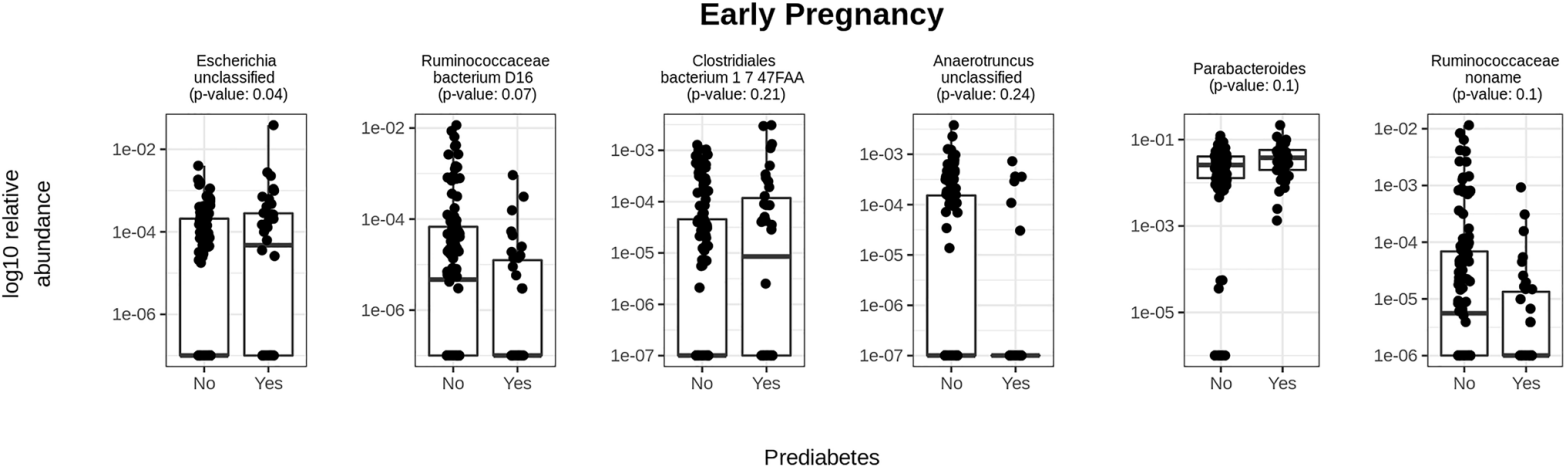
CLR transformed relative abundances of the two bacterial genera and four species with significant (*p* < 0.05) or borderline significant (FDR < 0.25) differences in early pregnancy between the women who developed prediabetes (*n* = 38) and those women who did not (*n* = 126). The significance was estimated with MaAsLin2. The following covariates were included in the model: prepregnancy BMI, dietary intake of PUFA

In early pregnancy, the relative abundance of *Parabacteroides*, *Escherichia unclassified* and *Clostridiales bacterium* 1_7_ 47FAA was higher and that of *Ruminococcaceae noname*, *Ruminococcaceae bacterium* D16 and *Anaerotruncus unclassified* was lower in women developing prediabetes as compared to those not developing. Only the bacterial species *Escherichia unclassified* differed between the groups below the significance level of FDR < 0.05. The other species were borderline significant with FDR < 0.25 (Fig. 2, Suppl. Table S3).

**Fig. 3:**
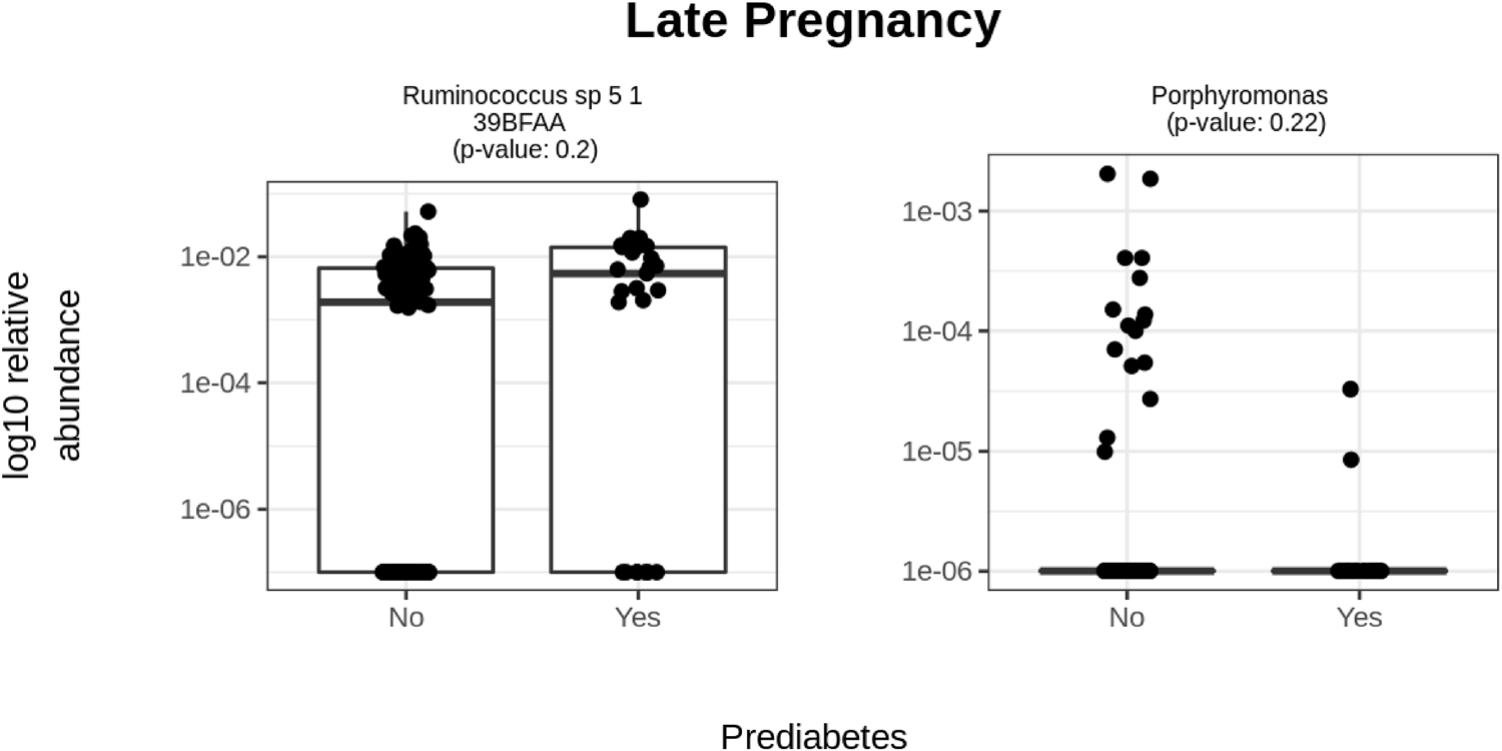
CLR transformed relative abundances of the one bacterial genus and one species differing borderline statistically significantly (FDR < 0.25) in late pregnancy between the women who developed prediabetes (*n* = 25) and those women who did not (*n* = 117). The significance was estimated with MaAsLin2. The following covariates were included in the model: prepregnancy BMI, dietary intake of PUFA

In late pregnancy, the relative abundance of *Ruminococcus* sp 5_1_39BFAA was higher in women developing prediabetes, while the relative abundance of *Porphyromonas* was lower in women developing prediabetes as compared to those not developing (FDR < 0.25 in all comparisons, MaAsLin2, Fig. 3, Supp. Table S4).

Regarding changes in species abundances from early to late pregnancy, no significant differences between the women developing prediabetes and those not were detected (Wilcoxon test; p > 0.05, data not shown).

### Associations between bacterial and clinical determinants of prediabetic women

**Fig. 4:**
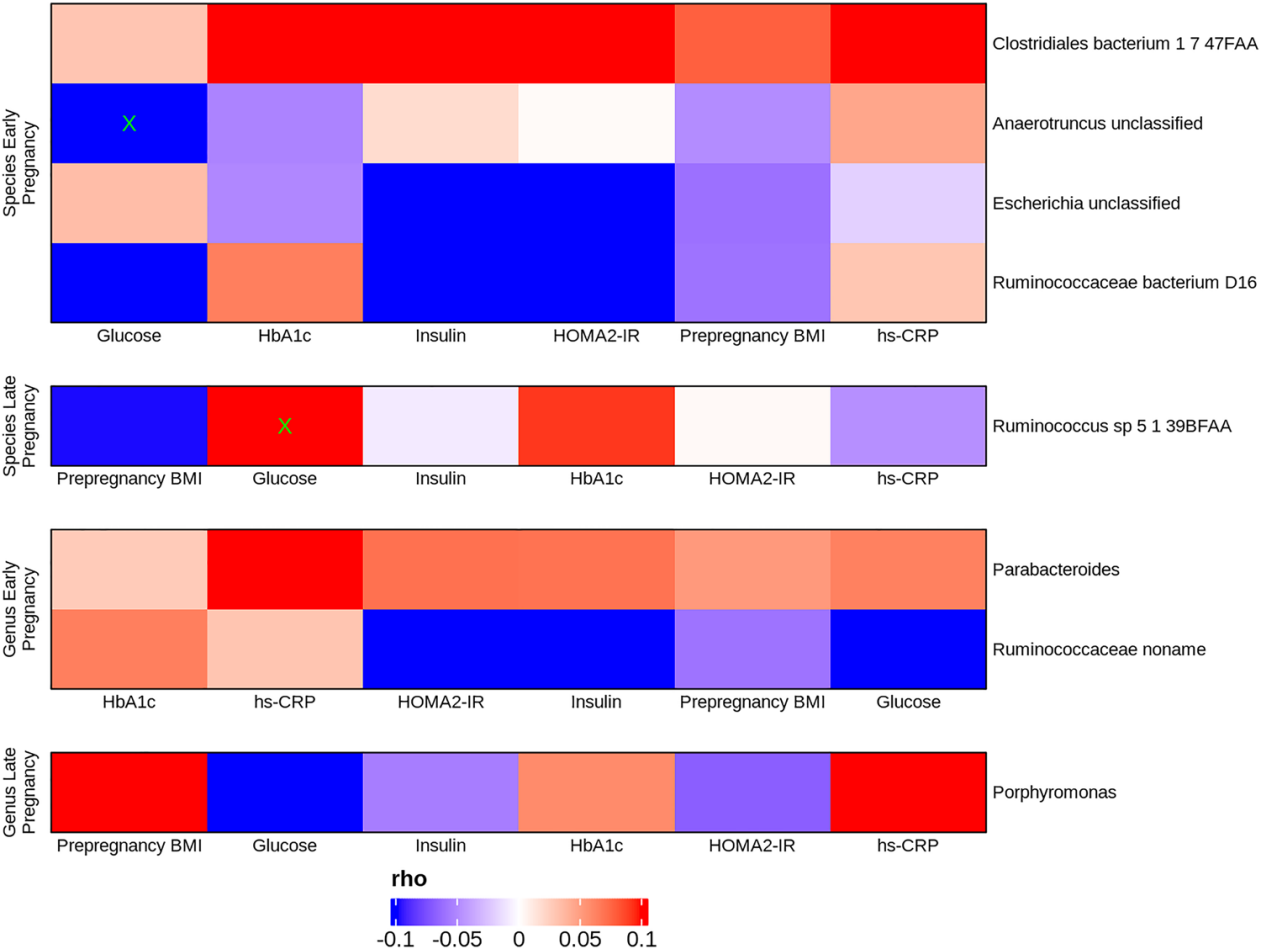
Heatmap describing the associations between bacterial species and genera that differ between the women who developed prediabetes and who did not in early and late pregnancy and hs-CRP, prepregnancy BMI and glycemic traits, these including fasting levels of glucose, fasting insulin, HbA_1c_, HOMA2-IR, determined at two-year postpartum in the whole study population (*n* = 142, except HbA_1c_: two missing values in early and late pregnancy, hs-CRP: one missing value in early pregnancy). Spearman correlation (rho); The associations were not significant; however, the borderline significant associations with FDR < 0.25 are denoted with X

Out of the eight bacterial species and genera that differed either significantly or borderline significantly in early and late pregnancy between the two groups, two bacterial species associated with fasting plasma glucose, namely *Anaerotruncus unclassified* inversely in early pregnancy and *Ruminococcus* sp 5_1_39BFAA directly in late pregnancy (FDR < 0.25, Fig. 4). Prepregnancy BMI values, fasting levels of insulin, HbA_1c,_ HOMA-IR or hs-CRP, were not associated with bacterial genera or species.

### Determinants of prediabetes at postpartum: functional profile of gut microbiota at species level during pregnancy

Out of the 511 functional pathways of gut microbiota in early and late pregnancy, 279 prevalent pathways were found to be present in 50% of the samples with a detection limit of 0. We focused on the analysis of these prevalent pathways in order to reduce multiple testing. We did not detect any significant differences in the abundance of prevalent pathways between the two groups with and without prediabetes (*p* > 0.05, linear model).

## Discussion

This study showed that gut microbiota composition in early and late pregnancy, but not gut microbiota diversity (*α*- and *β*-diversity) or function, contributes the prediabetes status at two-year postpartum. In particular, the abundances of putative short-chain fatty acids (SCFAs) producers, such as *Ruminococcaceae noname*, *Ruminococcaceae bacterium* D16, *Anaerotruncus unclassified* and *Porphyromonas,* were lower in women developing prediabetes. Furthermore, the blood glucose concentration was inversely associated with the abundances of potential SCFA-producing bacteria.

No previous studies exist which would have examined the relationship between the gut microbiota during pregnancy and the prediabetes at postpartum. We found that the bacteria that exhibited higher relative abundances in prediabetes were *Parabacteroides*, *Escherichia unclassified*, *Clostridiales bacterium* 1_7_ 47FAA and *Ruminococcus* sp 5_1_39BFAA, while lower abundances were detected for *Ruminococcaceae noname*, *Ruminococcaceae bacterium* D16, *Anaerotruncus unclassified* and *Porphyromonas*. There are previous studies reporting similar findings though the microbiota were assayed in non-pregnant subjects by 16S gene sequencing, i.e., increased *Escherichia coli* [6], enrichment of *Escherichia/Shigella* [26], higher *Ruminococcus* [27]. In one study applying metagenomics a higher *Escherichia coli* abundance was detected in prediabetic adults [9]. Nonetheless, there are some inconsistent findings, e.g., an enrichment of Ruminococcaceae [26, 27], analyzed by 16S gene sequencing. Other findings from previous studies involving subjects with prediabetes include changes in various bacteria [5, 26–28], including decreased *Akkermansia muciniphila* and lower Bacillota/Bacteroidota (formerly Bacteroidetes/Firmicutes) ratio as well as increased *Klebsiella* and *Dialister*. There is one report where the investigators used the 16S methodology and found no relation on gut microbiota in adults according to their prediabetes status [29]. Recently, Wu et al. [8] (2020) applied a metagenomics and detected a lower abundance of many butyrate-producing bacteria in prediabetic adults compared to their normoglycemic counterparts. Only one study has evaluated the gut microbiota before the onset of prediabetes as we did; in that report, *Klebsiella oxytoca* analyzed by metagenomics was lower in the non-pregnant individuals as compared to controls [10]. There are studies involving pregnant subjects which have focused on examining the impact of GDM on gut microbiota composition at postpartum which have and have not detected changes [30–32]. Crusell et al. [33] (2018) studied the gut microbiota in women in late pregnancy and eight-month postpartum in relation to GDM during pregnancy and found that the changes found in late pregnancy were still evident at eight-month postpartum. This may indicate that the aberrations found during pregnancy persist postpartum and may mediate the development of prediabetes. However, in our previous study the GDM status was not associated with gut bacterial species or diversity (12).

These present results, as well as those in the literature, suggest that the gut microbiota composition is linked to prediabetes. However, the detailed interpretation of our results and their comparison with previous findings are difficult for the following reasons, 1) many of these bacterial genera and species that we detected are still poorly documented in relation to prediabetes, 2) are inconsistent with previous reports or 3) have been reported at a lower or higher taxonomic rank than conducted here. It is noteworthy that our study is the first to follow the gut microbiota from pregnancy to postpartum; moreover, the metagenomics approach was used.

In our study, a functional analysis did not reveal significant results. In contrast, a few previous investigators have found that the functional potential of butyrate production is decreased [8] while there is an increase in other modules, e.g., bacterial secretion systems in prediabetic subjects [9], but the functional profile of the gut microbiota in these individuals has been rarely evaluated.

Mechanistically, four of the bacteria which were lower in the women developing prediabetes were close relatives to SCFA-producing bacteria, i.e., they can be considered as potential SCFA-producers, and these were members of the family Ruminococcaceae, including *Ruminococcaceae noname*, *Ruminococcaceae bacterium* D16 and *Anaerotruncus unclassified*, (as reviewed in [34]), and *Porphyromonas gingivalis*, which is in the bacterial genus *Porphyromonas* [35]. SCFAs can act via G-protein coupled receptors (GPCRs) which are located in various tissues, e.g., intestine, pancreas and adipose tissue (review [36]) and may thus influence glucose metabolism through insulin biosynthesis via the GPCRs located in pancreas. In agreement with the finding related to lower abundance of *Anaerotruncus unclassified* in women developing prediabetes, this bacteria was inversely correlated with glucose*.* The evidence for *Ruminococcus* sp 5_1_39BFAA in the onset of prediabetes was derived in two ways; first, a higher abundance was detected in women developing prediabetes and second, an association was detected with glucose levels. *Ruminococcus* sp 5_1_39BFAA is a member of the family Ruminococcaceae, and *Anaerotruncus unclassified* under family Oscillospiraceae which is a heterotypic synonym for Ruminococcaceae. Species which belong to the same genus with *Anaerotruncus unclassified* include *Anaerotruncus colihominis* which has been identified as an SCFA-producer, namely an acetic and butyric acid producer [37]. However, as discussed in the review of Louis et al. [34], not all bacteria that are members of the family *Ruminococcaceae* actually produce SCFAs, meaning that even though many bacteria may be related to each other, they may possess different characteristics and one needs to be cautious when interpreting the results. Thus, the bacterial species *Ruminococcus* sp 5_1_39BFAA, which was higher in the women developing prediabetes, might not be a beneficial SCFA-producer [34] which could explain our finding. In our study, *Escherichia unclassified* was higher in early pregnancy in women developing prediabetes and interestingly the pathogenic species belonging to the genus *Escherichia* produce toxins which may be involved in dysbiosis and further in disease progression (review [38]). All in all, the women developing prediabetes displayed a decreased abundance of potential beneficial SCFA-producing bacteria while there was an increased abundance of toxin-producing bacterium suggesting that the gut microbiota during pregnancy may pre-date the development of postpartum prediabetes.

The strengths of this report include the well-characterized study population with fecal samples available in both early and late pregnancy. Indeed, differences were found at both timepoints, although early in pregnancy the findings were more evident, at the level of FDR < 0.05. Our study participants were overweight and obese pregnant women, unfortunately representing a very typical population of pregnant women (currently 41.9% in Finland). Other strengths include our application of a robust metagenomics approach and bioinformatics tools for analyzing gut microbiota composition. Compared to 16S, a metagenomics approach offers a more accurate taxonomic resolution. One limitation of our study was the relatively small number of subjects who developed prediabetes, although in other studies with non-pregnant adults [5–7, 9, 10, 27–29, 39] the number of subjects has been similar or even smaller and it is noteworthy that this is the first to report the relation of the gut microbiota during pregnancy on the onset of prediabetes postpartum. However, due to the drop out, the sample size is relatively small as compared to the original sample size. Thus, we tested whether there are differences in the baseline characteristics between the women who were included in the study (*n* = 176) and who were not (*n* = 262) and there were minor differences, i.e., higher systolic and diastolic blood pressure as well as higher daily dietary intake of energy, fat, PUFA and fiber as well as percentage of family history of diabetes (yes = 20.6%, *n* = 175 vs 11.7%, *n* = 214, *p* = 0.03, Fisher’s exact test) in women who were included (Suppl. Table S5). Thus, it is possible that the included women were more likely to develop prediabetes since their parents had higher percentage of diabetes cases. Thus, further studies on the topic are called for.

## Conclusions

In summary, we identified specific taxonomic signatures in the composition of the gut microbiota during pregnancy that were determinants of the onset of prediabetes at two-year postpartum. However, neither the diversity nor the functional profile of the gut microbiota was associated with the onset of prediabetes.

## Supplementary Information

Below is the link to the electronic supplementary material.

Supplementary file1 (DOCX 352 KB)

Supplementary file2 (DOCX 22 KB)

Supplementary file3 (DOCX 91 KB)

## Data Availability

The datasets are not available due to the fact that they contain information that could compromise the privacy and consent of the participants.
