# Supplementary material for: Gut microbiota composition and function in pregnancy as determinants of prediabetes at two-year postpartum
Source: Acta Diabetol. 2023 Apr 28;60(8):1045–54. doi: 10.1007/s00592-023-02064-5 (PMC10289902; doi:10.1007/s00592-023-02064-5)
Supplement: Supplementary file 1 — Supplementary file1 (DOCX 352 KB) [file 592_2023_2064_MOESM1_ESM.docx]

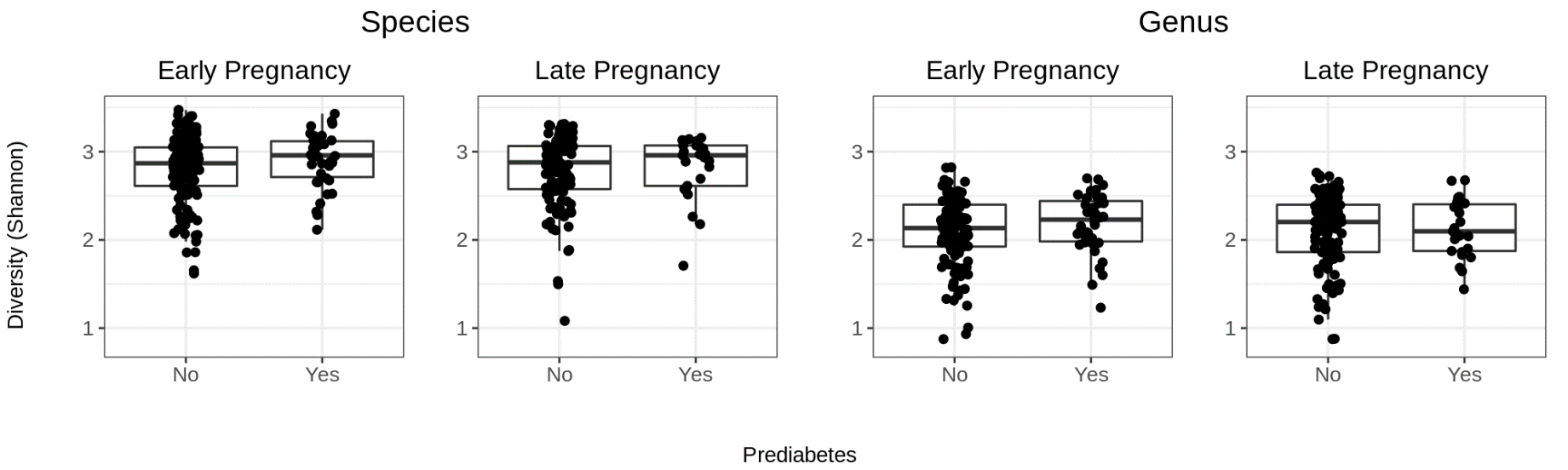


Supplementary Figure S1. α-Diversity (Shannon index) in early and late pregnancy in the women who developed prediabetes (early, n=38; late n=25) and those women who did not (early, n=126; late, n=117). No significant differences were observed (linear model, the following covariates were included in the model: prepregnancy BMI, dietary intake of PUFA)


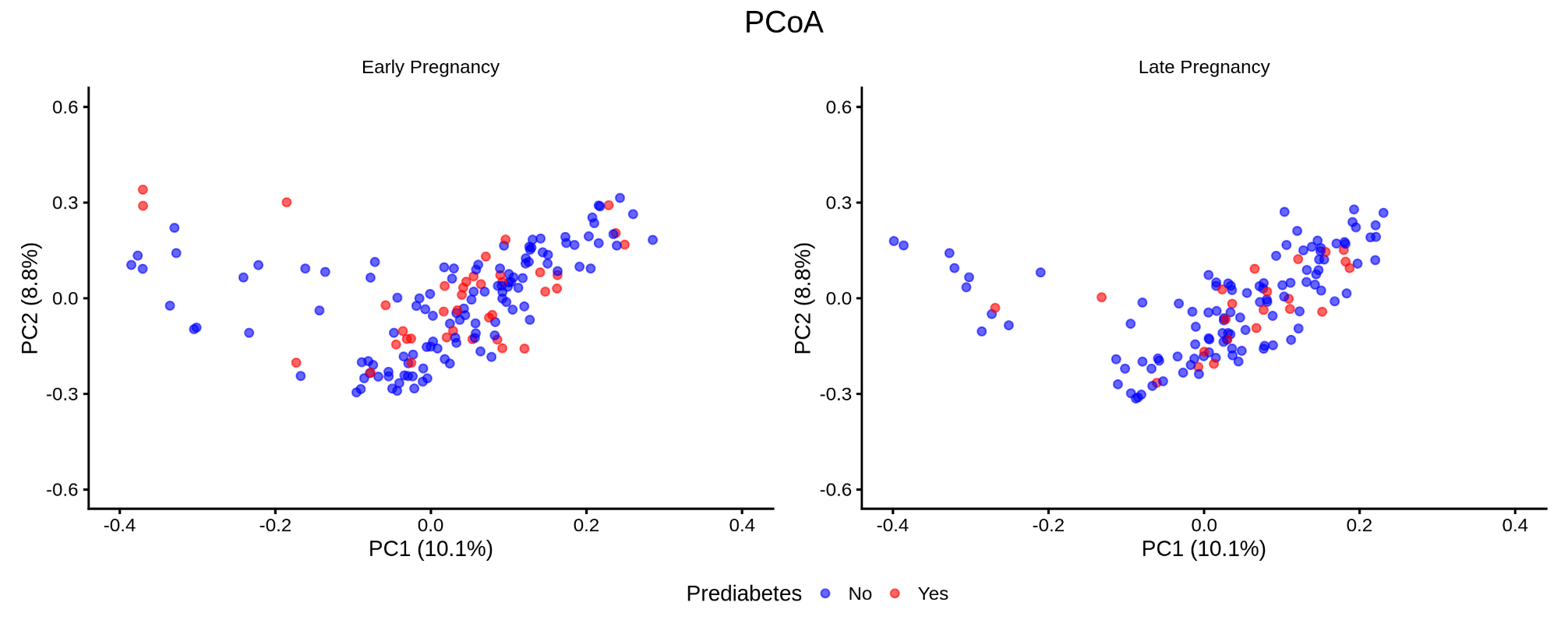


Supplementary Figure S2. PCoA in early and late pregnancy. Each point corresponds to a study participant and are colored to represent the following groups; red for the women who developed prediabetes (early, n=38; late, n=25) and blue for those who did not (early, n=126; late, n=117).


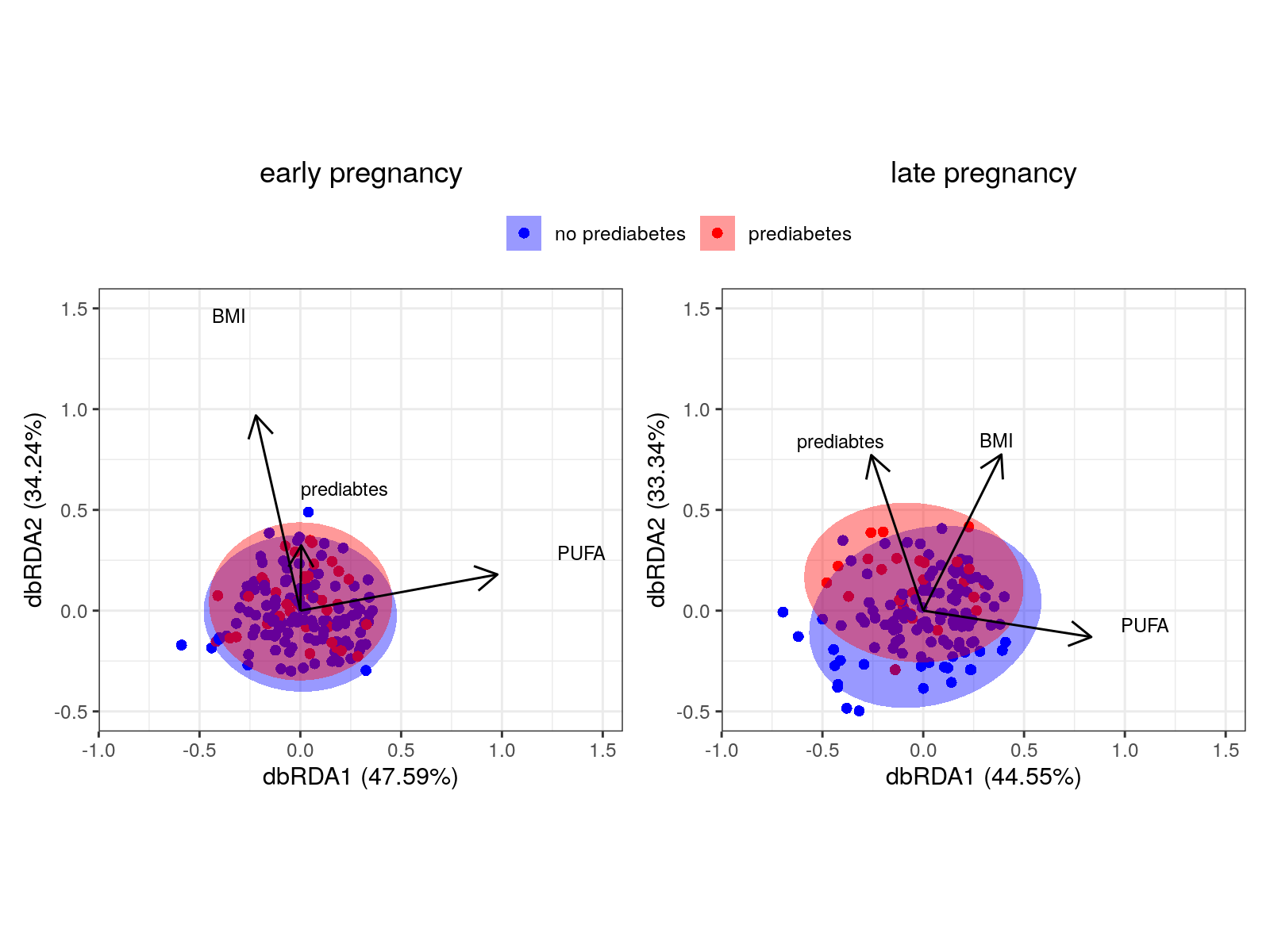


Supplementary Figure S3. Prediabetes risk groups are visualized with dbRDA (vegan::dbrda) separately for early and late pregnancy. Significance of PUFA at early pregnancy is confirmed also with dbRDA analysis, while BMI and prediabetes status are not significant (vegan::anova.cca).
