# Supplementary material for: Gut microbiota composition and function in pregnancy as determinants of prediabetes at two-year postpartum
Source: Acta Diabetol. 2023 Apr 28;60(8):1045–54. doi: 10.1007/s00592-023-02064-5 (PMC10289902; doi:10.1007/s00592-023-02064-5)
Supplement: Supplementary file 2 — Supplementary file2 (DOCX 22 KB) [file 592_2023_2064_MOESM2_ESM.docx]

**DNA extraction and metagenomics analyses**

DNA was extracted by using a GTX stool extraction kit and a fully automated GenoXTractmachine (Hain Lifescience). Before extraction, mechanical lysis was performed by bead-beating the samples in ceramic bead tubes with a MOBIO PowerLyzer 24Bench Top Bead-Based Homogenizer (MO BIO Laboratories, Inc., Carlsbad, CA).

Metagenomic sequencing was performed by Clinical Microbiomics (Denmark). The genomic DNA was randomly sheared into fragments of approximately 350 bp, which were used for library construction using NEBNext Ultra II Library Prep Kit for Illumina (New England Biolabs). The prepared DNA libraries were evaluated using Qubit V.2.0 fluorometer quantitation and Agilent 2100 Bioanalyzer for the fragment size distribution. Quantitative real-time PCR was used to determine the concentration of the final library before sequencing. The library was sequenced using 2×150 bp paired-end sequencing on an Illumina HiSeq-platform.

Quality control and preprocessing of raw FASTQ reads were performed using KneadData [1]. These steps included read trimming, adapter removal and separation of both rRNA sequences (SILVA V.128) and human host DNA (Hg38). KneadData was run with default settings except for specifying the above database versions.

The analysis of the microbial composition was performed using MetaPhlAn2 [2] V.2.6.0 with the default settings for paired-end reads. Pathway profiling was performed using the HUMAnN2 pipeline [3] V.0.11.1 in two steps. First, HUMAnN2 was run with ChocoPhlAn database V.0.1.1 and UniRef90 (created September 2016). Next, the files were renormalised to relative abundances using the helper script ‘humann2_renorm_table.py’ included in the HUMAnN2 distribution. The ChocoPhlAn and UniRef90 databases were downloaded using the supplied method (‘humann2_database--download uniref uniref90_diamond’, ‘humann2_database--download chocophlan full’).

References:

1. KneadData | The Huttenhower Lab.”. Available: http://huttenhower.sph.harvard.edu/kneaddata. Retrieved 7 October 2022

2. Segata N , Waldron L , Ballarini A , et al . Metagenomic microbial community profiling using unique clade-specific marker genes. Nat Methods 2012;9:811–4.doi:10.1038/nmeth.2066 pmid:http://www.ncbi.nlm.nih.gov/pubmed/22688413

3. Franzosa EA , McIver LJ , Rahnavard G , et al . Nat. Methods 2018;15:962–8.

**Dominant faecal bacterial taxa**

A total of 150 species and 55 genera were identified in the faecal samples collected during pregnancy. The four most abundant genera were *Bacteroides*, *Alistipes*, *Subdoligranulum* and *Eubacterium* in both early and late pregnancy (prevalences 100%, 99.4%, 100% in early and 100%, 98.6%, 100%, 99.3% in late pregnancy, respectively). The four most abundant species were *Subdoligranlum* *unclassified, Bacteroides* *unifromis, Faecalibacterium* *prausnitzii* and *Alistipes* *putredinis* in early pregnancy (prevalences 100%, 92.7%, 99.4%, 79.3% respectively) while *Subdoligranlum* *unclassified, Bacteroides* *unifromis*, *Prevotella copri* and *Alistipes putredinis* in late pregnancy (prevalences 100%, 94.4%, 33.8%, 83.3%, respectively) (the mean relative abundances and prevalences per group are presented in Suppl. Table S1-2).
