# Supplementary material for: Gut microbiota composition and function in pregnancy as determinants of prediabetes at two-year postpartum
Source: Acta Diabetol. 2023 Apr 28;60(8):1045–54. doi: 10.1007/s00592-023-02064-5 (PMC10289902; doi:10.1007/s00592-023-02064-5)
Supplement: Supplementary file 3 — Supplementary file3 (DOCX 91 KB) [file 592_2023_2064_MOESM3_ESM.docx]

Supplementary Table S1. The mean relative abundances and prevalences of all identified genera in all women in early (n=164) and late (n=142) pregnancy.

|  | early pregnancy | | | | late pregnancy | | | |
| --- | --- | --- | --- | --- | --- | --- | --- | --- |
|  | Mean Rel.Abund. (%) | | Prevalence (%) | | Mean Rel.Abund. (%) | | Prevalence (%) | |
|  | prediabetic | non-prediabetic | prediabetic | non-prediabetic | prediabetic | non-prediabetic | prediabetic | non-prediabetic |
| Bacteroides | 32.0 | 31.3 | 100.0 | 100.0 | 30.2 | 29.2 | 100 | 100.0 |
| Alistipes | 10.7 | 12.3 | 100.0 | 99.2 | 11.6 | 11.9 | 100 | 98.3 |
| Eubacterium | 8.4 | 8.1 | 100.0 | 100.0 | 5.4 | 8.9 | 100 | 99.1 |
| Subdoligranulum | 8.2 | 9.1 | 100.0 | 100.0 | 8.0 | 8.8 | 100 | 100.0 |
| Faecalibacterium | 5.2 | 5.8 | 100.0 | 99.2 | 6.8 | 5.9 | 100 | 99.1 |
| Parabacteroides | 4.6 | 2.9 | 100.0 | 91.3 | 3.7 | 3.3 | 92 | 94.0 |
| Prevotella | 3.5 | 5.5 | 39.5 | 50.8 | 7.4 | 6.4 | 40 | 53.0 |
| Ruminococcus | 3.4 | 2.9 | 97.4 | 92.1 | 4.3 | 3.1 | 100 | 93.2 |
| Roseburia | 2.8 | 3.0 | 100.0 | 100.0 | 2.4 | 3.0 | 100 | 99.1 |
| Barnesiella | 2.1 | 2.1 | 55.3 | 61.9 | 1.4 | 2.4 | 44 | 68.4 |
| Coprococcus | 2.0 | 1.2 | 89.5 | 91.3 | 2.0 | 1.4 | 92 | 88.0 |
| Dialister | 1.8 | 2.3 | 50.0 | 60.3 | 2.1 | 1.5 | 52 | 51.3 |
| Bifidobacterium | 1.4 | 1.4 | 97.4 | 96.8 | 0.9 | 1.9 | 100 | 97.4 |
| Paraprevotella | 1.3 | 0.6 | 31.6 | 27.0 | 1.3 | 0.5 | 32 | 25.6 |
| Blautia | 1.2 | 1.1 | 100.0 | 100.0 | 1.1 | 1.3 | 100 | 100.0 |
| Oscillibacter | 1.2 | 1.1 | 100.0 | 100.0 | 1.4 | 1.1 | 100 | 100.0 |
| Clostridium | 1.1 | 0.6 | 97.4 | 97.6 | 0.4 | 0.8 | 96 | 98.3 |
| Acidaminococcus | 0.9 | 0.2 | 13.2 | 13.5 | 0.4 | 0.2 | 8 | 14.5 |
| Sutterella | 0.9 | 1.0 | 52.6 | 52.4 | 1.5 | 1.0 | 64 | 53.0 |
| Odoribacter | 0.8 | 0.8 | 78.9 | 73.8 | 1.1 | 0.9 | 88 | 73.5 |
| Bacteroidales_noname | 0.7 | 0.9 | 63.2 | 60.3 | 0.8 | 0.7 | 76 | 63.2 |
| Lachnospiraceae_noname | 0.7 | 0.9 | 100.0 | 100.0 | 0.6 | 0.8 | 100 | 100.0 |
| Bilophila | 0.7 | 0.5 | 81.6 | 73.0 | 0.6 | 0.4 | 80 | 71.8 |
| Akkermansia | 0.6 | 1.0 | 76.3 | 71.4 | 0.9 | 1.2 | 76 | 70.9 |
| Escherichia | 0.5 | 0.2 | 76.3 | 77.8 | 0.4 | 0.2 | 68 | 82.1 |
| Streptococcus | 0.4 | 0.3 | 100.0 | 100.0 | 0.3 | 0.5 | 100 | 100.0 |
| Collinsella | 0.3 | 0.4 | 78.9 | 84.9 | 0.4 | 0.6 | 88 | 88.9 |
| Dorea | 0.3 | 0.4 | 100.0 | 98.4 | 0.5 | 0.5 | 100 | 99.1 |
| Phascolarctobacterium | 0.3 | 0.1 | 7.9 | 5.6 | 0.5 | 0.1 | 12 | 4.3 |
| Veillonella | 0.2 | 0.1 | 84.2 | 91.3 | 0.1 | 0.1 | 76 | 80.3 |
| Burkholderiales_noname | 0.2 | 0.1 | 60.5 | 56.3 | 0.1 | 0.1 | 68 | 59.0 |
| Parasutterella | 0.2 | 0.1 | 60.5 | 57.9 | 0.2 | 0.1 | 76 | 63.2 |
| Sutterellaceae_unclassified | 0.2 | 0.1 | 23.7 | 20.6 | 0.1 | 0.1 | 16 | 17.1 |
| Haemophilus | 0.2 | 0.1 | 73.7 | 78.6 | 0.0 | 0.1 | 40 | 58.1 |
| Coprobacter | 0.1 | 0.1 | 28.9 | 31.0 | 0.1 | 0.1 | 32 | 35.0 |
| Lactococcus | 0.1 | 0.2 | 65.8 | 73.0 | 0.0 | 0.2 | 60 | 60.7 |
| Clostridiales_noname | 0.1 | 0.0 | 52.6 | 46.8 | 0.0 | 0.0 | 48 | 40.2 |
| Anaerostipes | 0.1 | 0.1 | 92.1 | 88.1 | 0.0 | 0.1 | 92 | 85.5 |
| Peptostreptococcaceae_noname | 0.1 | 0.1 | 86.8 | 84.1 | 0.1 | 0.1 | 92 | 82.9 |
| Erysipelotrichaceae_noname | 0.1 | 0.1 | 81.6 | 77.0 | 0.1 | 0.1 | 76 | 81.2 |
| Holdemania | 0.1 | 0.0 | 94.7 | 91.3 | 0.0 | 0.0 | 96 | 88.9 |
| Megasphaera | 0.1 | 0.0 | 10.5 | 7.1 | 0.0 | 0.1 | 8 | 6.8 |
| Desulfovibrio | 0.1 | 0.0 | 13.2 | 20.6 | 0.0 | 0.0 | 20 | 19.7 |
| Megamonas | 0.0 | 0.1 | 2.6 | 6.3 | 0.1 | 0.1 | 4 | 6.0 |

The following genera were detected but left out of the table due to the low prevalence (relative abundance <0.01%) in the whole study population: *Adlercreutzia*, *Eggerthella*, *Porphyromonas*, *Lactobacillus*, *Clostridiaceae*_noname, *Flavonifractor*, *Pseudoflavonifractor*, *Anaerotruncus*, *Ruminococcaceae*_noname, *Catenibacterium* and *Coprobacillus*.

Supplementary Table S2. The mean relative abundances and prevalences of all identified species in all women in early (n=164) and late (n=142) late pregnancy.

|  | early pregnancy | | | | late pregnancy | | | |
| --- | --- | --- | --- | --- | --- | --- | --- | --- |
|  | Mean Rel.Abund. (%) | | Prevalence (%) | | Mean Rel.Abund. (%) | | Prevalence (%) | |
|  | prediabetic | non-prediabetic | prediabetic | non-prediabetic | prediabetic | non-prediabetic | prediabetic | non-prediabetic |
| Bacteroides_uniformis | 8.8 | 7.3 | 94.7 | 92.1 | 8.9 | 6.3 | 100 | 93.2 |
| Subdoligranulum_unclassified | 8.2 | 9.0 | 100.0 | 100.0 | 8.0 | 8.7 | 100 | 100.0 |
| Faecalibacterium_prausnitzii | 5.2 | 5.8 | 100.0 | 99.2 | 6.8 | 5.9 | 100 | 99.1 |
| Bacteroides_vulgatus | 4.8 | 3.4 | 86.8 | 81.0 | 6.0 | 3.8 | 88 | 84.6 |
| Alistipes_putredinis | 4.8 | 5.3 | 81.6 | 78.6 | 6.6 | 5.8 | 92 | 82.1 |
| Eubacterium_rectale | 4.5 | 3.9 | 89.5 | 93.7 | 3.0 | 4.6 | 92 | 93.2 |
| Bacteroides_dorei | 3.9 | 4.7 | 81.6 | 80.2 | 3.3 | 5.0 | 76 | 84.6 |
| Prevotella_copri | 3.5 | 5.3 | 18.4 | 31.0 | 7.4 | 6.3 | 36 | 33.3 |
| Parabacteroides_merdae | 2.7 | 1.6 | 76.3 | 61.9 | 2.5 | 1.7 | 72 | 65.8 |
| Alistipes_onderdonkii | 2.3 | 2.8 | 86.8 | 84.1 | 2.3 | 3.0 | 88 | 87.2 |
| Bacteroides_eggerthii | 2.2 | 1.2 | 18.4 | 25.4 | 1.1 | 1.2 | 24 | 22.2 |
| Bacteroides_fragilis | 2.2 | 1.4 | 68.4 | 65.1 | 1.2 | 1.1 | 72 | 68.4 |
| Ruminococcus_bromii | 2.2 | 1.7 | 68.4 | 53.2 | 2.2 | 2.0 | 76 | 58.1 |
| Barnesiella_intestinihominis | 2.1 | 2.1 | 55.3 | 61.9 | 1.4 | 2.4 | 44 | 68.4 |
| Bacteroides_ovatus | 1.9 | 2.3 | 92.1 | 94.4 | 1.2 | 2.1 | 96 | 95.7 |
| Dialister_invisus | 1.8 | 2.2 | 50.0 | 54.8 | 2.1 | 1.5 | 52 | 47.9 |
| Eubacterium_siraeum | 1.6 | 1.9 | 71.1 | 72.2 | 0.7 | 2.4 | 76 | 72.6 |
| Coprococcus_sp_ART55_1 | 1.6 | 0.8 | 28.9 | 21.4 | 1.6 | 0.8 | 56 | 26.5 |
| Alistipes_shahii | 1.5 | 1.4 | 78.9 | 71.4 | 1.3 | 1.2 | 80 | 75.2 |
| Bacteroides_caccae | 1.3 | 1.7 | 55.3 | 56.3 | 1.6 | 1.3 | 68 | 56.4 |
| Bacteroides_stercoris | 1.3 | 2.5 | 39.5 | 42.9 | 2.6 | 1.9 | 48 | 41.0 |
| Eubacterium_eligens | 1.3 | 1.6 | 78.9 | 84.1 | 1.2 | 1.3 | 84 | 83.8 |
| Alistipes_finegoldii | 1.2 | 1.1 | 86.8 | 87.3 | 0.8 | 0.8 | 88 | 85.5 |
| Oscillibacter_unclassified | 1.2 | 1.1 | 100.0 | 100.0 | 1.4 | 1.1 | 100 | 99.1 |
| Parabacteroides_unclassified | 1.1 | 0.6 | 36.8 | 40.5 | 0.5 | 0.8 | 48 | 44.4 |
| Bacteroides_cellulosilyticus | 1.0 | 1.5 | 50.0 | 53.2 | 1.0 | 1.3 | 52 | 58.1 |
| Bacteroides_massiliensis | 1.0 | 1.3 | 15.8 | 19.8 | 0.8 | 1.5 | 20 | 21.4 |
| Paraprevotella_unclassified | 1.0 | 0.4 | 31.6 | 27.0 | 1.0 | 0.3 | 32 | 25.6 |
| Roseburia_inulinivorans | 1.0 | 0.9 | 97.4 | 92.9 | 0.5 | 0.9 | 96 | 89.7 |
| Roseburia_hominis | 0.9 | 0.5 | 84.2 | 90.5 | 0.5 | 0.7 | 100 | 90.6 |
| Sutterella_wadsworthensis | 0.9 | 1.0 | 52.6 | 52.4 | 1.5 | 1.0 | 64 | 53.0 |
| Bifidobacterium_adolescentis | 0.8 | 0.9 | 76.3 | 69.8 | 0.6 | 1.1 | 76 | 71.8 |
| Bacteroides_faecis | 0.8 | 0.7 | 39.5 | 44.4 | 0.4 | 0.6 | 36 | 48.7 |
| Bacteroides_thetaiotaomicron | 0.8 | 0.7 | 65.8 | 62.7 | 0.5 | 0.5 | 72 | 65.8 |
| Odoribacter_splanchnicus | 0.8 | 0.8 | 71.1 | 70.6 | 1.1 | 0.9 | 84 | 67.5 |
| Ruminococcus_torques | 0.8 | 0.8 | 100.0 | 99.2 | 0.7 | 0.9 | 96 | 96.6 |
| Bacteroidales_bacterium_ph8 | 0.7 | 0.9 | 63.2 | 60.3 | 0.8 | 0.7 | 76 | 63.2 |
| Acidaminococcus_unclassified | 0.7 | 0.2 | 13.2 | 12.7 | 0.2 | 0.2 | 8 | 12.8 |
| Bilophila_unclassified | 0.7 | 0.5 | 81.6 | 72.2 | 0.5 | 0.4 | 80 | 71.8 |
| Eubacterium_hallii | 0.6 | 0.5 | 97.4 | 93.7 | 0.3 | 0.4 | 100 | 95.7 |
| Ruminococcus_lactaris | 0.6 | 0.6 | 65.8 | 55.6 | 1.0 | 0.5 | 80 | 58.1 |
| Akkermansia_muciniphila | 0.6 | 1.0 | 76.3 | 71.4 | 0.9 | 1.2 | 76 | 70.9 |
| Bacteroides_intestinalis | 0.5 | 0.6 | 28.9 | 22.2 | 0.3 | 0.4 | 24 | 23.9 |
| Parabacteroides_distasonis | 0.5 | 0.5 | 71.1 | 62.7 | 0.4 | 0.6 | 64 | 65.0 |
| Roseburia_intestinalis | 0.5 | 1.0 | 78.9 | 73.0 | 1.0 | 1.0 | 68 | 73.5 |
| Bifidobacterium_longum | 0.4 | 0.3 | 89.5 | 90.5 | 0.2 | 0.6 | 80 | 91.5 |
| Clostridium_sp_L2_50 | 0.4 | 0.1 | 5.3 | 4.0 | 0.1 | 0.2 | 8 | 4.3 |
| Eubacterium_ventriosum | 0.4 | 0.2 | 55.3 | 57.1 | 0.1 | 0.2 | 36 | 59.8 |
| Roseburia_unclassified | 0.4 | 0.6 | 52.6 | 47.6 | 0.4 | 0.4 | 48 | 34.2 |
| Ruminococcus_sp_5_1_39BFAA | 0.4 | 0.5 | 52.6 | 50.0 | 1.0 | 0.4 | 72 | 54.7 |
| Collinsella_aerofaciens | 0.3 | 0.4 | 73.7 | 82.5 | 0.4 | 0.6 | 84 | 86.3 |
| Bacteroides_xylanisolvens | 0.3 | 0.3 | 47.4 | 69.8 | 0.3 | 0.2 | 64 | 74.4 |
| Paraprevotella_clara | 0.3 | 0.2 | 28.9 | 24.6 | 0.4 | 0.1 | 32 | 22.2 |
| Alistipes_sp_AP11 | 0.3 | 0.3 | 28.9 | 32.5 | 0.4 | 0.2 | 40 | 34.2 |
| Alistipes_sp_HGB5 | 0.3 | 0.5 | 7.9 | 10.3 | 0.0 | 0.2 | 8 | 6.8 |
| Streptococcus_thermophilus | 0.3 | 0.2 | 89.5 | 81.0 | 0.2 | 0.3 | 88 | 78.6 |
| Ruminococcus_obeum | 0.3 | 0.2 | 100.0 | 99.2 | 0.3 | 0.3 | 100 | 97.4 |
| Coprococcus_comes | 0.3 | 0.2 | 84.2 | 86.5 | 0.3 | 0.3 | 84 | 84.6 |
| Phascolarctobacterium_succinatutens | 0.3 | 0.1 | 7.9 | 5.6 | 0.5 | 0.1 | 12 | 4.3 |
| Escherichia_coli | 0.3 | 0.1 | 65.8 | 72.2 | 0.4 | 0.2 | 60 | 75.2 |
| Bacteroides_coprocola | 0.2 | 0.5 | 7.9 | 13.5 | 0.4 | 0.5 | 8 | 15.4 |
| Bacteroides_finegoldii | 0.2 | 0.5 | 15.8 | 19.8 | 0.2 | 0.3 | 16 | 19.7 |
| Bacteroides_plebeius | 0.2 | 0.4 | 5.3 | 11.1 | 0.0 | 0.2 | 0 | 12.8 |
| Bacteroides_sp_2_1_22 | 0.2 | 0.0 | 2.6 | 1.6 | 0.0 | 0.1 | 4 | 1.7 |
| Parabacteroides_johnsonii | 0.2 | 0.1 | 13.2 | 15.9 | 0.2 | 0.1 | 16 | 17.9 |
| Alistipes_unclassified | 0.2 | 0.5 | 13.2 | 17.5 | 0.0 | 0.4 | 4 | 14.5 |
| Clostridium_bolteae | 0.2 | 0.1 | 78.9 | 72.2 | 0.1 | 0.2 | 60 | 65.0 |
| Clostridium_leptum | 0.2 | 0.1 | 89.5 | 80.2 | 0.1 | 0.2 | 92 | 87.2 |
| Clostridium_nexile | 0.2 | 0.0 | 7.9 | 10.3 | 0.0 | 0.0 | 0 | 9.4 |
| Ruminococcus_gnavus | 0.2 | 0.1 | 52.6 | 57.9 | 0.0 | 0.1 | 72 | 52.1 |
| Dorea_longicatena | 0.2 | 0.3 | 92.1 | 84.9 | 0.3 | 0.4 | 92 | 88.9 |
| Lachnospiraceae_bacterium_1_1_57FAA | 0.2 | 0.4 | 57.9 | 57.1 | 0.3 | 0.3 | 56 | 47.0 |
| Burkholderiales_bacterium_1_1_47 | 0.2 | 0.1 | 60.5 | 56.3 | 0.1 | 0.1 | 68 | 59.0 |
| Parasutterella_excrementihominis | 0.2 | 0.1 | 60.5 | 57.9 | 0.2 | 0.1 | 76 | 63.2 |
| Haemophilus_parainfluenzae | 0.2 | 0.1 | 73.7 | 78.6 | 0.0 | 0.1 | 40 | 58.1 |
| Bifidobacterium_bifidum | 0.1 | 0.1 | 34.2 | 29.4 | 0.1 | 0.1 | 32 | 35.0 |
| Bacteroides_clarus | 0.1 | 0.0 | 10.5 | 16.7 | 0.2 | 0.1 | 12 | 16.2 |
| Coprobacter_fastidiosus | 0.1 | 0.1 | 28.9 | 31.0 | 0.1 | 0.1 | 32 | 35.0 |
| Alistipes_indistinctus | 0.1 | 0.2 | 52.6 | 47.6 | 0.1 | 0.2 | 44 | 53.0 |
| Alistipes_senegalensis | 0.1 | 0.1 | 50.0 | 47.6 | 0.1 | 0.1 | 52 | 47.0 |
| Lactococcus_lactis | 0.1 | 0.2 | 65.8 | 72.2 | 0.0 | 0.2 | 60 | 59.8 |
| Streptococcus_salivarius | 0.1 | 0.1 | 94.7 | 92.9 | 0.0 | 0.1 | 92 | 97.4 |
| Clostridium_sp_ATCC_BAA_442 | 0.1 | 0.0 | 2.6 | 0.8 | 0.0 | 0.0 | 0 | 3.4 |
| Eubacterium_ramulus | 0.1 | 0.1 | 73.7 | 69.8 | 0.2 | 0.1 | 76 | 70.9 |
| Anaerostipes_hadrus | 0.1 | 0.0 | 86.8 | 87.3 | 0.0 | 0.1 | 92 | 84.6 |
| Coprococcus_catus | 0.1 | 0.1 | 76.3 | 60.3 | 0.1 | 0.1 | 80 | 63.2 |
| Dorea_formicigenerans | 0.1 | 0.1 | 94.7 | 92.9 | 0.1 | 0.1 | 96 | 93.2 |
| Lachnospiraceae_bacterium_3_1_46FAA | 0.1 | 0.2 | 89.5 | 92.9 | 0.2 | 0.2 | 92 | 89.7 |
| Lachnospiraceae_bacterium_5_1_63FAA | 0.1 | 0.1 | 97.4 | 91.3 | 0.1 | 0.1 | 100 | 91.5 |
| Lachnospiraceae_bacterium_7_1_58FAA | 0.1 | 0.1 | 100.0 | 97.6 | 0.1 | 0.1 | 100 | 94.9 |
| Lachnospiraceae_bacterium_8_1_57FAA | 0.1 | 0.1 | 26.3 | 27.8 | 0.0 | 0.1 | 32 | 30.8 |
| Ruminococcus_callidus | 0.1 | 0.1 | 42.1 | 39.7 | 0.2 | 0.1 | 64 | 36.8 |
| Megasphaera_unclassified | 0.1 | 0.0 | 10.5 | 6.3 | 0.0 | 0.0 | 8 | 5.1 |
| Veillonella_parvula | 0.1 | 0.0 | 65.8 | 68.3 | 0.0 | 0.0 | 40 | 56.4 |
| Veillonella_unclassified | 0.1 | 0.1 | 84.2 | 89.7 | 0.0 | 0.0 | 76 | 80.3 |
| Bilophila_wadsworthia | 0.1 | 0.0 | 76.3 | 67.5 | 0.0 | 0.1 | 76 | 59.0 |
| Desulfovibrio_piger | 0.1 | 0.0 | 13.2 | 7.9 | 0.0 | 0.0 | 8 | 8.5 |
| Escherichia_unclassified | 0.1 | 0.0 | 55.3 | 49.2 | 0.0 | 0.0 | 36 | 54.7 |
| Bifidobacterium_pseudocatenulatum | 0.0 | 0.0 | 21.1 | 26.2 | 0.0 | 0.1 | 24 | 32.5 |
| Bacteroides_salyersiae | 0.0 | 0.2 | 5.3 | 9.5 | 0.0 | 0.2 | 4 | 11.1 |
| Bacteroides_sp_3_2_5 | 0.0 | 0.1 | 0.0 | 3.2 | 0.0 | 0.1 | 0 | 2.6 |
| Parabacteroides_goldsteinii | 0.0 | 0.1 | 7.9 | 15.1 | 0.0 | 0.1 | 4 | 16.2 |
| Prevotella_stercorea | 0.0 | 0.1 | 0.0 | 3.2 | 0.0 | 0.1 | 0 | 3.4 |
| Streptococcus_parasanguinis | 0.0 | 0.0 | 86.8 | 81.7 | 0.0 | 0.1 | 72 | 83.8 |
| Coprococcus_eutactus | 0.0 | 0.1 | 7.9 | 5.6 | 0.0 | 0.2 | 12 | 8.5 |
| Clostridium_bartlettii | 0.0 | 0.1 | 71.1 | 70.6 | 0.1 | 0.0 | 80 | 76.9 |
| Subdoligranulum_sp_4_3_54A2FAA | 0.0 | 0.1 | 13.2 | 15.1 | 0.0 | 0.1 | 4 | 17.1 |
| Eubacterium_biforme | 0.0 | 0.1 | 0.0 | 13.5 | 0.0 | 0.1 | 0 | 16.2 |
| Dialister_succinatiphilus | 0.0 | 0.1 | 0.0 | 4.8 | 0.0 | 0.1 | 0 | 4.3 |
| Megamonas_unclassified | 0.0 | 0.0 | 2.6 | 6.3 | 0.0 | 0.1 | 4 | 6.0 |

The following species were detected but left out of the table due to the low prevalence (relative abundance <0.01%) in the whole study population: *Bifidobacterium_animalis*, *Bifidobacterium_catenulatum*, *Adlercreutzia_equolifaciens*, *Collinsella_intestinalis*, *Collinsella_tanakaei*, *Collinsella_unclassified*, *Eggerthella_unclassified*, *Bacteroides_coprophilus*, *Bacteroides_nordii*, *Paraprevotella_xylaniphila, Lactobacillus_acidophilus*, *Lactobacillus_ruminis*, *Clostridiaceae_bacterium_JC118*, *Clostridium_asparagiforme*, *Clostridium_citroniae*, *Clostridium_clostridioforme*, *Clostridium_hathewayi*, *Clostridium_symbiosum*, *Clostridiales_bacterium_1_7_47FAA*, *Flavonifractor_plautii*, *Pseudoflavonifractor_capillosus*, *Eubacterium_sp_3_1_31*, *Dorea_unclassified*, *Lachnospiraceae_bacterium_1_4_56FAA*, *Lachnospiraceae_bacterium_2_1_58FAA*, *Lachnospiraceae_bacterium_3_1_57FAA_CT1*, *Oscillibacter_sp_KLE_1745*, *Peptostreptococcaceae_noname_unclassified*, *Anaerotruncus_colihominis*, *Anaerotruncus_unclassified*, *Ruminococcaceae_bacterium_D16*, *Catenibacterium_mitsuokai*, *Coprobacillus_unclassified*, *Eubacterium_cylindroides*, *Holdemania_filiformis*, *Holdemania_unclassified*, *Megamonas_funiformis*, *Megamonas_hypermegale*, *Veillonella_atypica* and *Desulfovibrio_desulfuricans.*

Supplementary Table S3. Relative abundances of the two bacterial genera and four species with statistically (p<0.05) and borderline statistically significant (FDR<0.25) differences in early pregnancy between the women who developed prediabetes (n=38) and those women who did not (n=126). The significance was estimated with MaAsLin2. The following covariates were included in the model: prepregnancy BMI, dietary intake of PUFA.

| Taxa | Rank | Mean Relative Abundance (%) | | Adjusted p-value | | | Effect Size | | |
| --- | --- | --- | --- | --- | --- | --- | --- | --- | --- |
|  |  | Yes prediabetes | No prediabetes | Prediabetes^a^ | PUFA^b^ | BMI^c^ | Prediabetes^a^ | PUFA^b^ | BMI^c^ |
| *Escherichia unclassified* | Species | 0.1 | 0.02 | 0.036 | 0.584 | 0.771 | 1.910 | -0.498 | -0.207 |
| *Ruminococcaceae bacterium* D16 | Species | 0 | 0.04 | 0.072 | 0.584 | 0.758 | -2.476 | -0.551 | 0.265 |
| *Clostridiales bacterium* 1 7 47FAA | Species | 0.03 | 0.01 | 0.211 | 0.739 | 0.771 | 1.168 | -0.233 | -0.153 |
| *Anaerotruncus unclassified* | Species | 0 | 0.02 | 0.239 | 0.176 | 0.739 | -1.471 | 0.534 | 0.215 |
| *Parabacteroides* | Genera | 5 | 3 | 0.103 | 0.981 | 0.959 | 0.470 | 0.005 | 0.018 |
| *Ruminococcaceae noname* | Genera | 0 | 0.04 | 0.103 | 0.721 | 0.853 | -2.468 | -0.549 | 0.261 |
| ^a^Prediabetes status at two years’ postpartum | | | | | | | | | |
| ^b^Dietary intake of polyunsaturated fatty acids | | | | | | | | | |
| ^c^Prepregnancy body mass index | | | | | | | | | |

Supplementary Table S4. The relative abundances of the one bacterial genus and the one species differing borderline statistically significantly (FDR<0.25) in late pregnancy between the women who developed prediabetes (n=25) and those women who did not (n=117). The significance was estimated with ANCOMBC. The following covariates were included in the model: prepregnancy BMI, dietary intake of PUFA.

| Taxa | Rank | Mean Relative Abundance (%) | | Adjusted p-value | | | Effect Size | | |
| --- | --- | --- | --- | --- | --- | --- | --- | --- | --- |
|  |  | Yes prediabetes | No prediabetes | Prediabetes^a^ | BMI^b^ | PUFA^c^ | Prediabetes^a^ | BMI^b^ | PUFA^c^ |
| *Ruminococcus* sp 5 1 39BFAA | Species | 0.96 | 0.42 | 0.197 | 0.606 | 0.932 | 0.943 | -0.226 | -0.077 |
| *Porphyromonas* | Genera | 0 | 0.01 | 0.221 | 0.039 | 0.893 | -5.083 | 1.010 | 0.196 |
| ^a^Prediabetes status at two years’ postpartum | | | | | | | | | |
| ^b^Dietary intake of polyunsaturated fatty acids | | | | | | | | | |
| ^c^Prepregnancy body mass index | | | | | | | | | |

Supplementary Table S5. The baseline clinical characteristics which differed statistically significantly between women were included in the study and who were not (drop outs).

| Clinical characteristics | Included | Not included | n | P value |
| --- | --- | --- | --- | --- |
| Age (y)^a^ | 31.4 ± 4.6 | 30.0 ± 4.5 | 176/261 | 0.002 |
| Systolic blood pressure (mmHg)^a^ | 118.9 ± 10.7 | 115.7 ± 9.9 | 175/260 | 0.002 |
| Diastolic blood pressure (mmHg)^a^ | 78.0 ± 8.4 | 75.6 ± 8.1 | 175/260 | 0.003 |
| Family history of diabetes (n, %)^b^ |  |  | 175/214 | 0.032 |
| Yes | 36, 20.6 | 25, 11.7 |  |  |
| No | 130, 74.3 | 170, 79.4 |  |  |
| Does not know | 9, 5.1 | 19, 8.9 |  |  |
| Dietary intake |  |  |  |  |
| Energy (kJ)^a^ | 8426.4 ± 2028 | 7940.8 ± 1883.6 | 171/249 | 0.012 |
| Fat (g)^c^ | 80.8 (64.2-98.0) | 74.8 (59.9-90.4) | 171/249 | 0.014 |
| Polyunsaturated fat (g)^a^ | 13.1 ± 5.2 | 12.1 ± 4.4 | 171/249 | 0.045 |
| Fiber (g)^c^ | 20.7 (15.2-26.1) | 18.0 (14.1-23.3) | 171/249 | 0.002 |

^a^Independent-Samples T-test

^b^Fisher's exact test

^c^Mann-Whitney
